# Exosomal miR-126 as a circulating biomarker in non-small-cell lung cancer regulating cancer progression

**DOI:** 10.1038/s41598-017-15475-6

**Published:** 2017-11-10

**Authors:** Franco Grimolizzi, Federica Monaco, Francesca Leoni, Massimo Bracci, Sara Staffolani, Cristiana Bersaglieri, Simona Gaetani, Matteo Valentino, Monica Amati, Corrado Rubini, Franca Saccucci, Jiri Neuzil, Marco Tomasetti, Lory Santarelli

**Affiliations:** 1 Department of Clinical Sciences, Section of Biochemistry, Biology and Physics, Via Tronto 10/a, 60020 Ancona, Italy; 2 Department of Clinical and Molecular Sciences, Section of Occupational Medicine, Via Tronto 10/a, 60020 Ancona, Italy; 3 Department of Biomedical Sciences and Public Health, Section of Anatomical Pathology, Polytechnic University of Marche, Via Tronto 10/a, 60020 Ancona, Italy; 4 School of Medical Science, Griffith University, Southport, Qld 4222 Australia; 5 Institute of Biotechnology, Czech Academy of Sciences, Prague-West, 25242 Czech Republic

## Abstract

Lung cancer is one of the leading causes of cancer-related deaths. It is diagnosed mostly at the locally advanced or metastatic stage. Recently, micro RNAs (miRs) and their distribution in circulation have been implicated in physiological and pathological processes. In this study, miR-126 was evaluated in serum, exosome and exosome-free serum fractions in non-small cell lung cancer (NSCLC) patients at early and advanced stages, and compared with healthy controls. Down-regulation of miR-126 was found in serum of advanced stage NSCLC patients. In healthy controls, circulating miR-126 was equally distributed between exosomes and exosome-free serum fractions. Conversely, in both early and advanced stage NSCLC patients, miR-126 was mainly present in exosomes. Different fractions of miR-126 in circulation may reflect different conditions during tumour formation. Incubation of exosomes from early and advanced NSCLC patients induced blood vessel formation and malignant transformation in human bronchial epithelial cells. On the other hand, exosome-enriched miR-126 from normal endothelial cells inhibited cell growth and induces loss of malignancy of NSCLC cells. These findings suggest a role of exo-miRs in the modulation of the NSCLC microenvironmental niche. Exosome-delivered miRs thus hold a substantial promise as a diagnostics biomarker as well as a personalized therapeutic modality.

## Introduction

Lung cancer is a leading cause of cancer deaths among both men and women, and non-small-cell lung cancer (NSCLC) accounts for 80% of lung cancer cases^1^. Although recent advances have furthered the understanding of lung cancer pathogenesis, the survival rate of patients has not improved significantly^2^. Therefore, much effort has been focused on identification of new diagnostic markers and molecules involved in the disease development that could become targets for new therapeutic strategies.

Exosomes represent a specific subtype of extracellular vesicles (EV), ranging between 30 and 100 nm in diameter. They are released by several types of cells under both physiological and pathological conditions. Tumour-derived exosomes, interacting with other cells of the tumour microenvironment, modulate tumour progression, the angiogenic switch, metastasis, and immune escape^3^. Exosomes contain proteins, lipids and mRNA, and are especially rich in microRNAs (miRs)^4,5^. Exosomal miRs (exo-miRs) present a recently discovered means of cell-to-cell communication mechanism involved in establishment and maintenance of tumour microenvironment and the metastatic niche^6–8^. Although extracellular miRs can be transported within the circulation in association with proteins (i.e. Ago2, HDL, and other RNA-binding proteins), exo-miRs are remarkably stable compared to miRs that are not contained in exosomes^9,10^.

MiR-126 has been reported to be delivered to the tumour environment^5^. We and others have shown that during cancer progression, miR-126 is down-regulated, with ensuing changes in the expression and activity of metabolism-related factors^11–15^. MiR-126 is an endothelial-specific miR, which is expressed at low levels in NSCLC patients^12,16,17^, and its re-expression inhibits cell proliferation *in vitro* and tumour growth *in vivo* by targeting EGFL7^18^. Given that circulating miRs are differentially regulated and selectively packaged in exosomes, we evaluated the distribution of miR-126 in the circulation of NSCLC patients at early and advanced stages of the disease, and compared the level of the miR with healthy subjects. We then examined the possibility that exo-miR-126 discriminates NSCLC patients at different stages from controls, as well as that it affects tumour formation and progression.

## Results

### Levels of miR-126 in serum compartments of NSCLC patients

**Figure 1:**
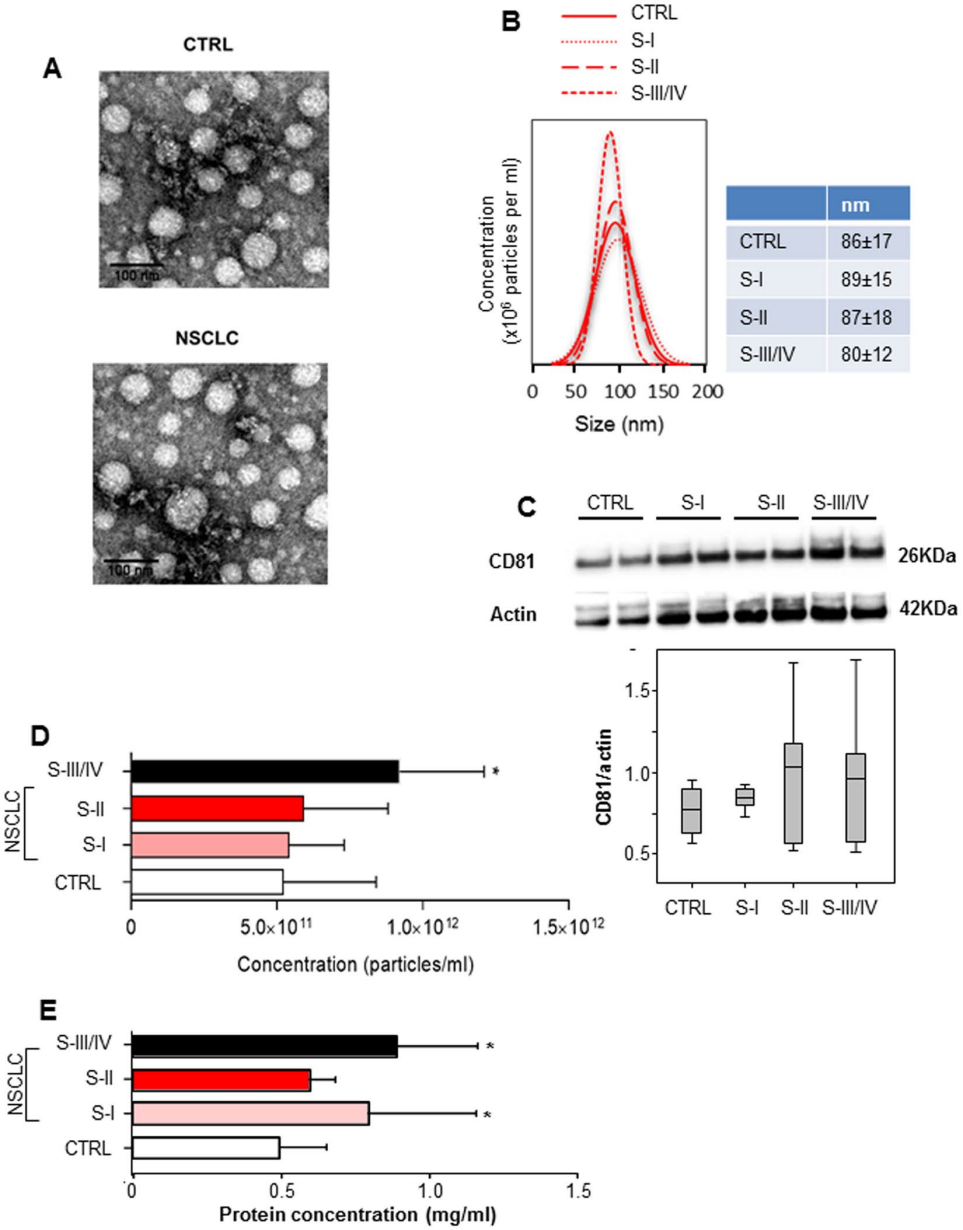
Characterization and quantification of exosome levels in serum samples from healthy controls and NSCLC patients. (**A**) Transmission electron microscopy (TEM) showing exosomes isolated from serum of healthy subjects (CTRL), and from non-small cell lung cancer (NSCLC) patients. Scale bars represent 100 μm. (**B**) Average histogram was plotted to show the overall size distribution of exosomes of CTRL and NSCLC patients at different tumour stages (Stage I, Stage II, Stage III/IV) using the Nanoparticle Tracking Analysis (NTA) system. (**C**) CD81 immunoblot of isolated exosomes (upper panel) and densitometry evaluation (lower panel). Bar graphs show concentration of exosomes calculated by the NTA system (**D**) and protein quantification (**E**). The data and images are derived from three or more independent experiments. Statistical analyses were performed using one-way analysis of variance (ANOVA), with Tukey post-hoc analysis. The symbol “*” indicates significant differences compared to control with *p* < 0.05.

From March 2015 to March 2017, 45 patients with NSCLC and 31 control subjects (CTRL) were enrolled. According to the 7^th^ lung cancer TNM classification, NSCLC patients have been included in three stage groups: Stage I group (TIaN0, TIbN0, TIIaN0), Stage II group (TIaN1, TIIbN0, TIIbN1, TIIIN0, TIIINxMx), Stage III/IV group (TIIbN2, TIVN0, TIIIN2, TIaN2, TIbN2, TIIaN2, TIIbN2, TxN3M0, TIVN0M1b, TIIIN0M1a, TIIINxM1a). The demographic characteristics are summarized in Table 1. Initially, exosomes were isolated from serum of the subjects and their level in the circulation of patients of various tumour stages evaluated by several methods. Isolated exosomes were visualized by transmission electron microscopy (TEM), their size distribution was evaluated using nanoparticle tracking analysis (NTA) technology, and the concentration was estimated by the number of particles and exosomal protein level. The presence of CD81, a specific exosome marker, was confirmed by western blotting. The size distribution based on the NTA analysis was in agreement with the microscopy images. Based on the particle number, protein and CD81 levels, advanced NSCLC patients (Stage III/IV group) showed higher levels of circulating exosomes compared to other groups (Fig. 1A,B). On the other hand, no relevant differences in concentration, size and number of exosomes between healthy subjects and early stage NSCLC patients (Stage I/II groups) were observed (Fig. 1C–E).

**Table 1:** Demographic and histopathological characteristics of the study populations.

	NSCLC Patients (n = 45)		Control subjects (n = 31)	
	mean ± SD	%	mean ± SD	%
Age (yrs)	71 ± 11		48 ± 17	
Gender (n°)				
male	32	71	23	74
female	13	29	8	26
Current or former smoker (n°)				
no	6	13	20	65
yes	25	56	7	22
ex	14	31	4	13
**NSCLC Stages**				
	**Stage I**	**Stage II**	**Stage III/IV**	
Age (mean ± SD)	(n = 16)	(n = 10)	(n = 19)	
Gender (n°)	76 ± 7	58 ± 9	72 ± 9	
male				
female	11 (69%)	7 (70%)	14 (74%)	
Current or former smoker (n°)	5 (31%)	3 (30%)	5 (26%)	
no	2 (12%)	1 (10%)	3 (16%)	
yes	12 (75%)	4 (40%)	9 (47%)	
ex	2 (13%)	5 (50%)	7 (37%)

**Figure 2:**
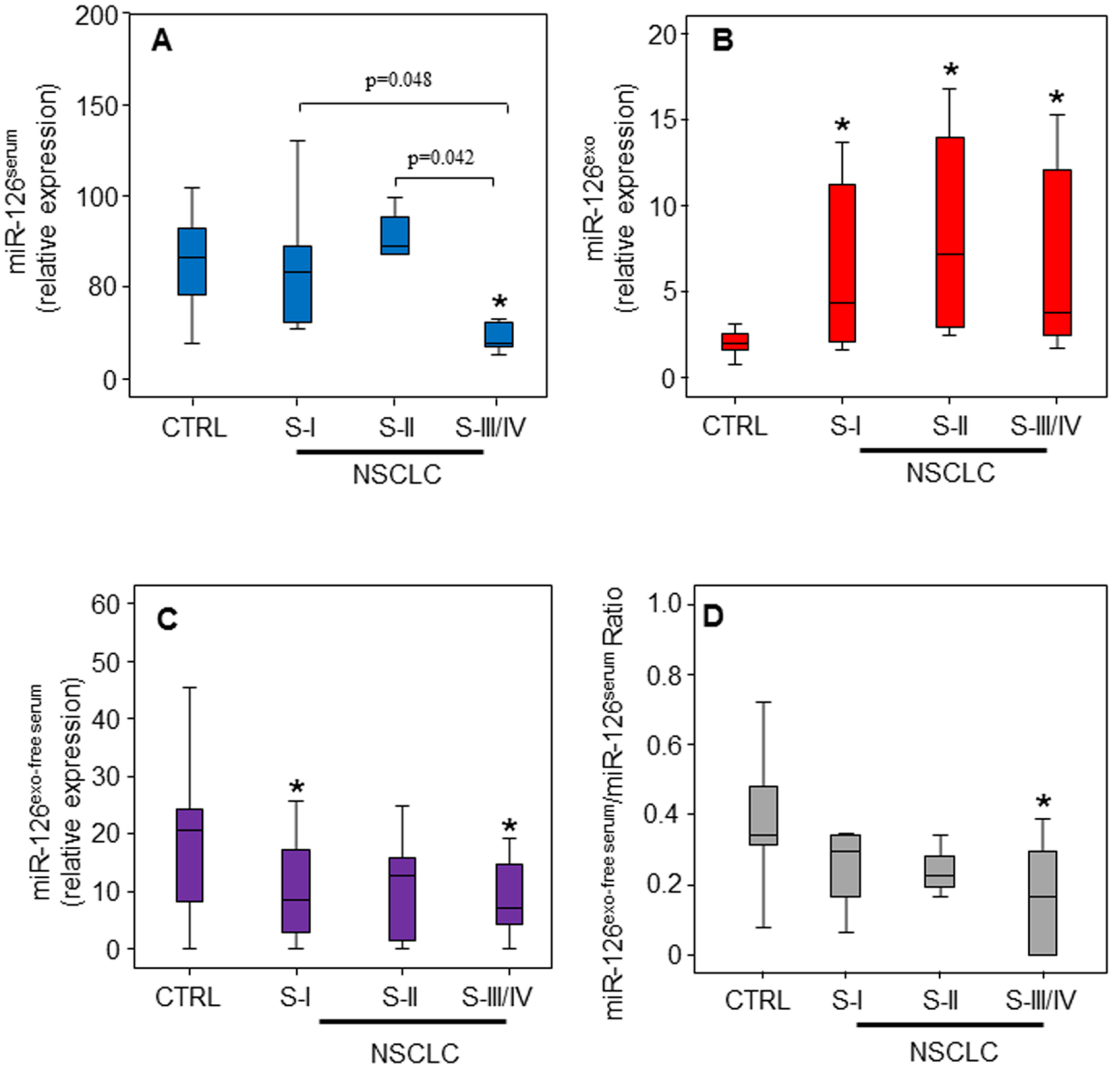
Distribution of miR-126 in serum, exosomes, and exosome-free serum in relation to tumour stage. Relative levels of miR-126 in serum (**A**), exosomes (**B**) and exosomes-free serum (**C**) in healthy control group (CTRL) and NSCLC patients of individual. (**D)** The ratio of circulating miR-126 in exosomes-free serum and serum of CTRL subjects and NSCLC patients at different tumour stages is shown. Statistical analyses were performed using one-way analysis of variance (ANOVA), with Tukey post-hoc analysis. The symbol “*” indicates significant differences compared to control with *p* < 0.05.

We next studied the level of miR-126 in three compartments: total serum, exosomes (exo), and exosome-free serum (exo-free serum), in the study groups (CTRL and NSCLC- Stage I, Stage II, Stage III/IV groups). We found that at early stages, the level of serum miR-126 was comparable in early NSCLC patients and control subjects, while it was significantly down-regulated in advanced NSCLC patients (Fig. 2A). Both at early and advanced stages, miR-126 was mainly present in exosomes (Fig. 2B). The increased level of miR-126 in exosomes paralleled the decrease of miR-126 content in the exosome-free serum fraction (Fig. 2C). The distribution of miR-126 in the exosomal and extra-exosomal compartments was also evaluated. As shown in Fig. 2D, the miR-126^exo-free serum^/miR-126^serum^ ratio decreased in NCSLC compared to healthy controls with a significant low ratio value at late stage, thus suggesting that at this stage miR-126 was encapsulated within exosomes rather than associated with circulating proteins.

### Exosomal miR-126 as a circulating biomarker for NSCLC progression

**Figure 3:**
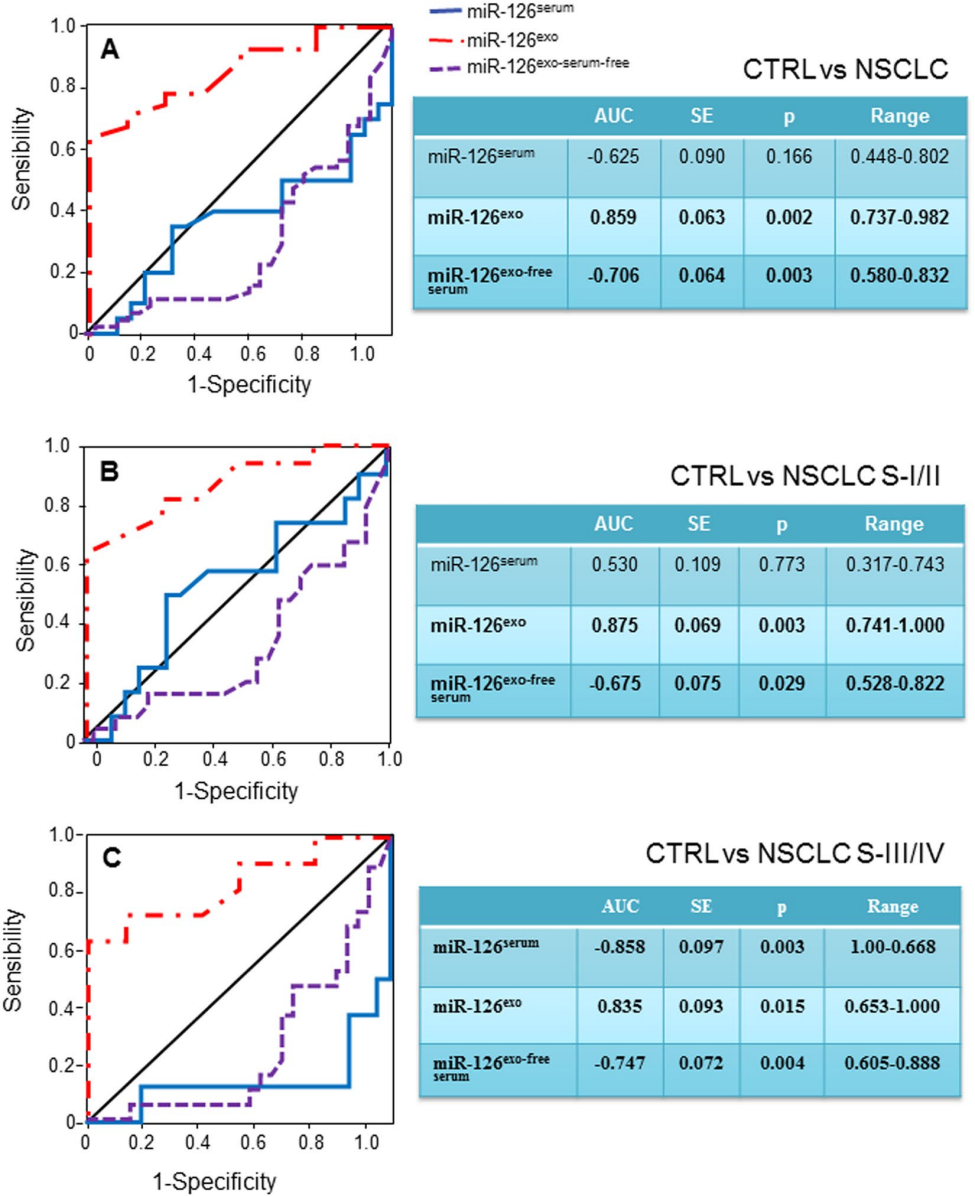
Biomarker performance in patients with NSCLC. Receiver-operating analysis of miR-126 detected in serum, exosomes and exosome-free serum. The areas under the receiver operating curves (AUC) were generated using miR-126 expression in serum, exosomes and exosome-free serum, discriminating healthy subjects (CTRL) from NSCLC patients at early (NSCLC-S-I/II) and advanced stages (NSCLC-S-III/IV). Differences with p < 0.05 were considered statistically significant.

The different distribution of exo-miR-126 in the serum from cancer patients versus healthy individuals has revealed important differences in relation to tumour progression, highlighting a possible use of this miR as a disease biomarker. Therefore, the performance of circulating miR-126 to distinguish patients affected by NSCLC at different stages of disease progression from controls was evaluated. The ROC analysis shows that serum miR-126 differentiated neither controls from NSCLC patients in general nor NSCLC at early stages, while discriminating the disease at advanced stage. Notably, exo-miR-126 significantly discriminated NSCLC patients at early from those at advanced tumour stages (Fig. 3A–C).

### Exo-miR-126 from endothelial cells suppresses NSCLC tumorigenic function

**Figure 4:**
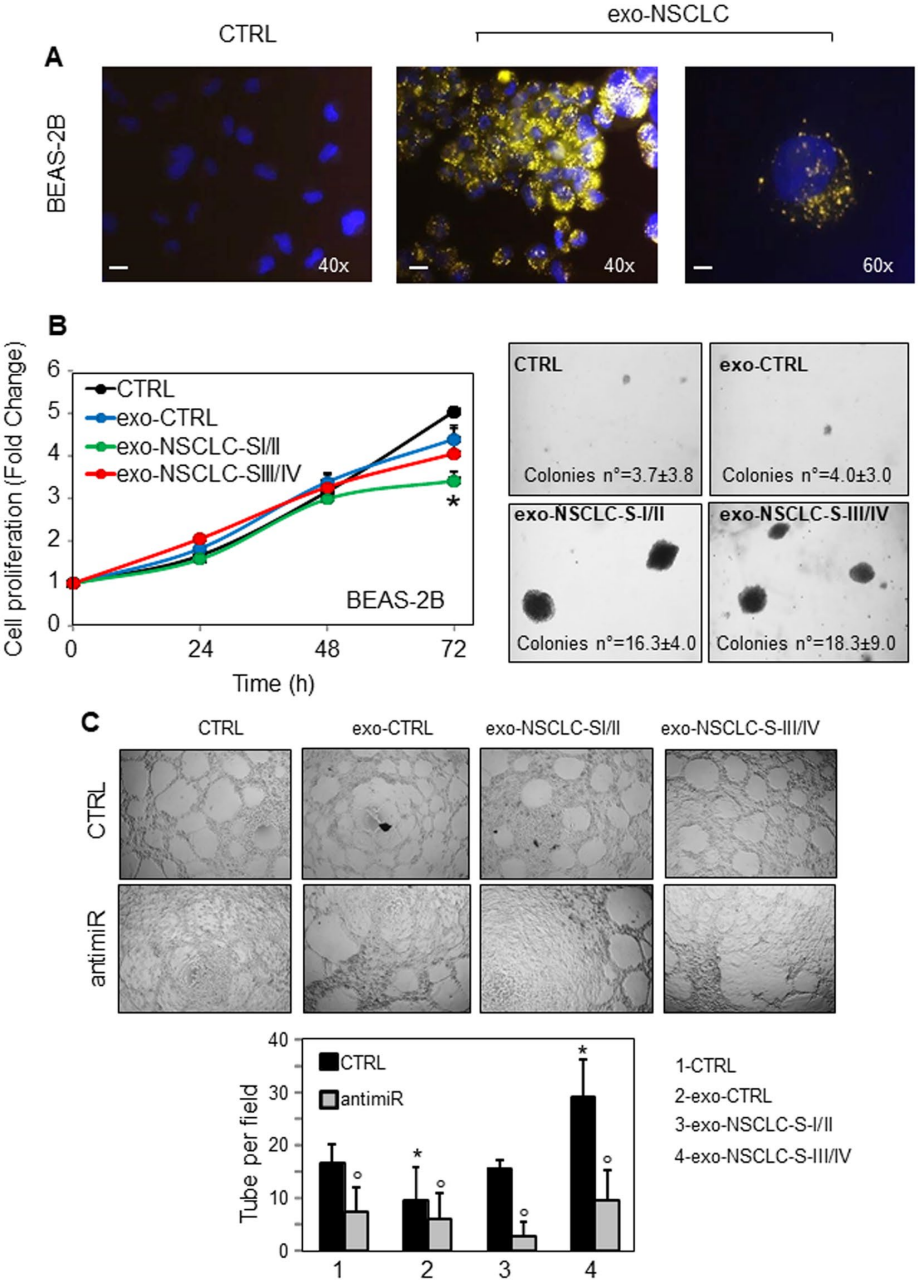
NSCLC-derived exosomes at early and advanced stages affect cell proliferation and induce angiogenesis and cell transformation. (**A**) Representative fluorescence image of NSCLC-derived exosome (exo-NSCLC) uptake by human bronchial epithelial cells (BEAS-2B). BEAS-2B cells, cultured in exosome-depleted serum, were incubated with acridine orange (AO)-labelled exo-NSCLC (20 µg/ml), and their internalization was visualised using a fluorescent microscope (Zeiss, Axiocam MRc5; magnification 400–600 x). Scale bar for all images equals 100 μm. (**B**) Cell proliferation (left panel) and colony-forming assay (right-panel) of BEAS-2B cells incubated with healthy subject-derived exosomes (CTRL), and NSCLC-derived exosome at early (exo-NSCLC Stage I/II) and advanced (exo-NSCLC Stage III/IV) stages. Representative images at 3 months after treatment are shown (magnification 100 x). (**C**) EAhy926 cells and their anti-miR-transfected counterparts were seeded in 96-well plates with 50 μl of Matrigel per well so that suspension of 200 μl of complete medium with 2 × 10^4^ cells were added to each well. Control cultures as well as those incubated with exosomes from healthy subjects (CTRL) and NSCLC-derived exosome (20 μg/ml) at early (exo-NSCLC Stage I/II) and advanced (exo-NSCLC Stage III/IV) stages were evaluated by counting the number of complete tubes connecting points of individual polygons of the capillary network in a light microscope at 24 h (lower panel). The data and images are relative of three independent experiments. Comparisons among groups were determined by one-way ANOVA with Tukey post-hoc analysis. The symbol “*” indicates significant differences compared with control, and the symbol “°” indicates significant between CTRL and anti-miR, with *p* < 0.05.

MiR-126 has been reported to act as a tumour suppressor and metastasis inhibitor in cancer, while inducing cell proliferation and angiogenesis in non-tumorigenic cells^19^. It was observed that exosome secretion maintains cellular homeostasis by removing toxic DNA fragments from cells^20^. Therefore, we can hypothesize that cancer cells may eliminate miR-126 via the exosomal pathway, with ensuing uptake of miR by recipient cells to induce distal transformation and angiogenesis, promoting cancer progression. To address these hypothesizes, NSCLC-derived exosomes (exo-NSCLC) were incubated with non-tumorigenic epithelial and endothelial cells, and proliferation, transformation and vessel formation were evaluated. As shown in Fig. 4A, exosomes from NSCLC patients were taken up at high rates by the recipient BEAS-2B cells, eliciting their decreased proliferation with concomitant transformation (Fig. 4B). Further, exo-NSCLC induced angiogenesis, which was inhibited by specific anti-miR, supporting the role of miR-126 in the regulation of this process (Fig. 4C).

**Figure 5:**
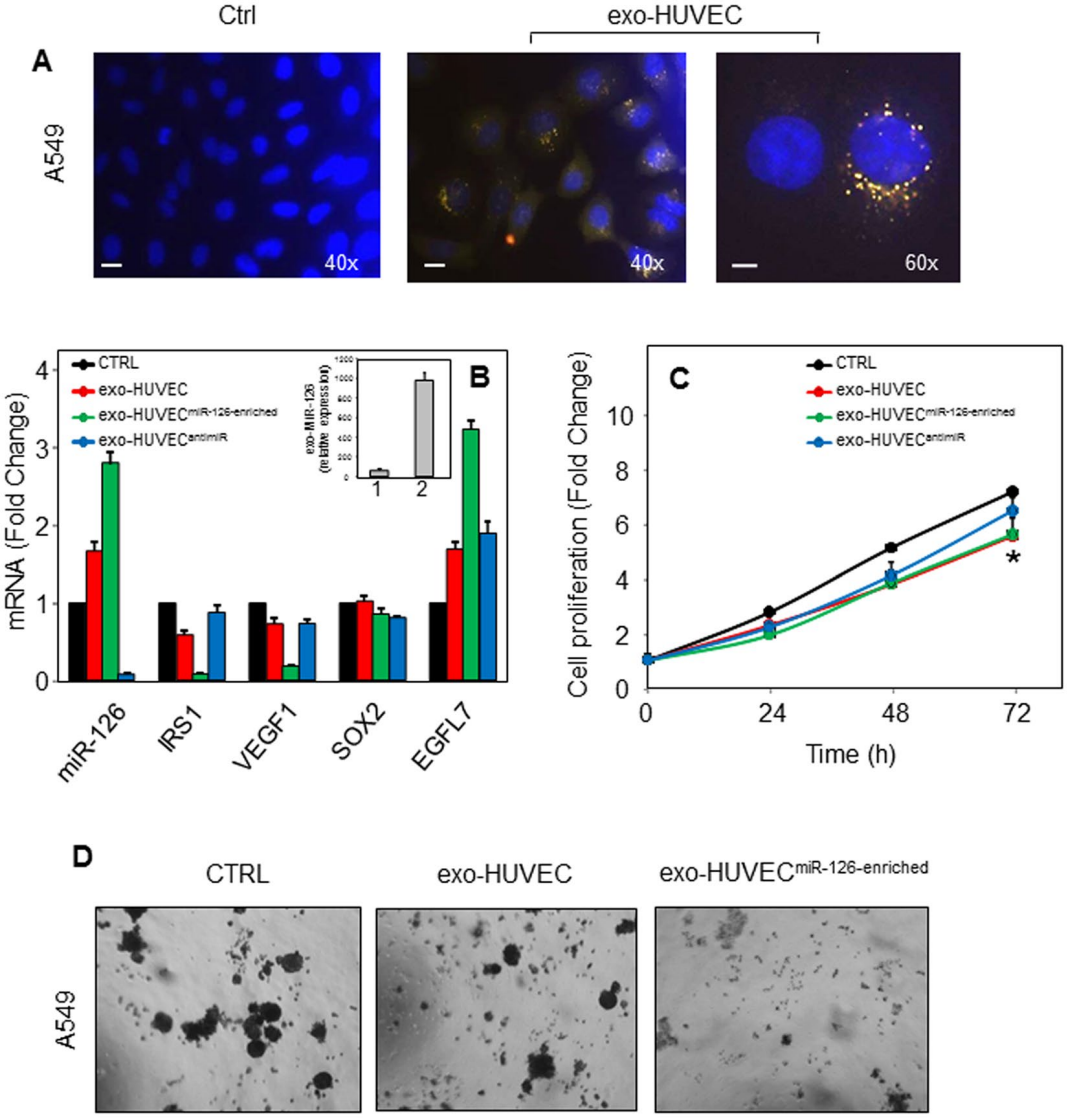
Exosomal transfer of miR-126 from endothelial cells inhibits cell proliferation and cell transformation. (**A**) Representative fluorescence images of uptake of HUVEC-derived exosomes by A549 cells. A549 cells, cultured in exosome-depleted serum, were incubated with acridine orange (AO)-labelled HUVEC-derived exosomes (20 µg/ml) for 4 h and their internalization was visualised by fluorescent microscopy (magnification 400–600 x). Scale bar for all images equals 100 μm. (**B**) Gene expression of miR-126 targets insulin receptor substrate1 (IRS1), vascular endothelial growth factor (VEGF) -1, sex determining region Y-box 2 (SOX2), and EGF-like domain-containing protein 7 (EGFL7) after incubation of A549 cells with HUVEC-derived exosomes and miR-126-enriched exosomes derived from HUVECs grown under glucose deprivation (exo-HUVEC^miR-126-enriched^), with or without anti-miR. The level of miR-126 in HUVEC-derived exosomes (1) and miR-126-enriched HUVEC-derived exosomes (2) is shown in the insert. Cell proliferation (**C**) and colony-forming assay (**D**) in A549 cells after treatment with HUVEC-derived exosomes and miR-126-enriched HUVEC-derived exosomes. The data shown are mean values ± S.D. derived from three independent experiments. Representative images taken 3 months after treatment are shown (magnification 100 x). Comparisons among groups were determined by one-way ANOVA with Tukey post-hoc analysis. The symbol “*” indicates significant differences with *p* < 0.05.

MiR-126 is highly expressed in endothelial cells, and endothelial-derived exosomes contain high levels of miR-126^21^. Therefore, endothelial-derived exosomes isolated from human umbilical vein endothelial cells (HUVECs) (HUVEC exosomes), and miR-126-enriched exosomes from HUVECs cultured under condition of glucose deprivation, were incubated with NSCLC cells, and exosome uptake, miR-126 content, and expression of its targets were evaluated. In addition, proliferation rates of NSCLC cells and malignancy following exosome treatment were also investigated. Uptake of exosomes by NSCLC recipient cells was observed after 4 h incubation with HUVEC exosomes previously stained with acridine orange (Fig. 5A). We found that exosomes readily interacted with NSCLC A549 cells, leading to increased miR-126 level in malignant cells, which was related to the exosomal content of miR-126 (Fig. 5B, insert). Increased miR-126 was accompanied by suppression of miR-126 targets, such as IRS1 and VEGF, which were restored by anti-miR treatment. On the other hand, SOXO2 and EGFL7 were not affected by HUVEC exosomes (Fig. 5B). As shown in Fig. 5C, HUVEC exosomes significantly inhibited cancer cell proliferation independently of their miR-126 levels, and reduced colony formation and growth in soft agar (Fig. 5D).

## Discussion

Tumour cells secrete exosomes to modify the local microenvironment, which then promotes intercellular communication and metastasis. During stromal microenvironment remodelling, endothelial cells (ECs) are activated from a quiescent state leading to pathological angiogenesis^21^. MiR-126 has been reported to be mainly produced by ECs, delivered to the surrounding tumour milieu, and to regulate angiogenesis^16,22^. As previously found, a significant reduction of serum miR-126 was observed at advanced stages of tumour formation^12,14^. Similar results were observed by Lin *et al*., showing that miR-126 level was stable at the early stages of NSCLC, while it was significantly down-regulated at Stage IV, suggesting a link to metastasis formation^17^. Others found low levels of circulating miR-126 both at early and advanced stages of the neoplasia^23–25^, indicating that the method of normalization used may affect the results. Here, we found an increase of exosomal miR-126 in NSCLC patients both at early and late stages of the disease (*cf* Fig. 2) as previously found^26^. NSCLC tumours eliminate miR-126 by exosome, thus altering the pattern of distribution of miR-126 in circulation.

Multiple cell types secrete exosomes. It this may be difficult to discern the cellular origin of miR-126-loaded exosomes. There is a hypothesis that tumour cells use exosomes to transport genetic information including miRs to surrounding cells, whereby supporting tumour growth and progression^27^. Therefore, pathological conditions can promote characteristic changes to the vesicle cargo and contribute to secretion of miR-126-enriched exosomes into circulation. MiR-126 has been widely reported as a tumour suppressor in lung cancer due to targeting IRS1, EGFL7, Crk, and SLC7A513^18^, or by means of inhibiting angiogenesis and lung metastasis during lung cancer^28,29^. Therefore, it is not surprising that the extracellular imbalance observed can be triggered by tumour cells to drive the progression of neoplasia.

The different exosomal levels of miR-126 between NSCLC patients and controls suggests its role as a biomarker of the disease progression. In the present study, we found that exo-miR-126 differentiated healthy controls and NSCLC patients even at early stages, more significantly that with respect to miR-126 detected in serum (*cf* Fig. 3), supporting its use as a biomarker for NSCLC patients to detect the malignancy in its early stages. This is consistent with the notion that exo-miRNAs have recently emerged as potentially promising diagnostic and prognostic biomarkers in cancer and other diseases^30^. Recently, serum-derived exo-miRs such as exo-miR-21 and exo-miR-155 were found to be significantly upregulated in serum exosomes in recurrent NSCLC-bearing animals *vs*. non-tumour- or primary tumour-bearing animals^31^. Moreover, exosome-encapsulated miR-21 was significantly increased in patients with oesophageal squamous cell carcinoma, correlating with advanced tumour stage, positive lymph node status and metastasis^32^. Thus, exo-miRs hold considerable promise as vehicles for diagnosis of cancer and, potentially, for its management.

Exosomes play an important role in intercellular communication by acting as natural carriers of biologically active molecules between cells, thus modulating and reprogramming recipient cells. Tumour-derived exosomes function as pro-tumorigenic factors that can mediate intercellular communication in the tumour microenvironment and can also contribute to cancer progression^33^. Here, we demonstrated for the first time that transfer of exosomal cargo from patients affected by NSCLC at early and advanced stages of the disease made the tumour microenvironment beneficial for cancer cells, inducing distal malignant transformation and angiogenesis (*cf* Fig. 4). Consistent with this, exosomes from pleural effusion of malignant mesothelioma (MM) patients have been shown to modulate the angiogenic and metastatic potential of mesothelioma cells^34^. Further, exosomes from serum of breast cancer patients, but not those from healthy controls, were shown to induce transformation of non-tumorigenic epithelial cells, suggesting a potential role for exosome in tumorigenesis^35^. As observed previously in MM, we show here that NSCLC-derived exosomes carrying high levels of miR-126 significantly enhanced HUVEC tube formation compared to the vehicle control. Silencing of miR-126 by anti-miR markedly suppressed angiogenesis, further supporting its role in vessel formation. Intriguingly, miR-126 is thought to possess dual angiogenesis regulatory properties. On one hand, it can inhibit angiogenesis and maintain the quiescent endothelial phenotype. On the other hand, upregulation of miR-126 can activate endothelial progenitor cells (EPCs) and ECs, contributing to formation of new blood vessels by a mechanism that involves SPRED1 inhibition^36^. However, despite the pro-angiogenic role of miR-126, other factors delivered by exosome, such as EGFR, may induce vessel formation^20,37^.

The ability of exosomes to transport functionally active cargo, thus affecting recipient cells, might be used as a tool in cancer therapy. Therefore, we exposed cancer cells to exosomes from ECs (HUVECs) carrying miR-126, and have found that the tumour-suppressive miR-126 secreted from ECs could be taken up by recipient cancer cells, exerting angiogenic and cancer cell growth inhibitory function (*cf* Fig. 5). EC-derived miR-126 was found compartmentalised mainly to circulating exosomes. Certain cells, such as cardiomyocytes, increase their exosome secretion under glucose starvation conditions. Moreover, cardiomyocytes-derived exosomes modulate their miR cargo in a glucose-dependent manner. *In vitro* experiments revealed that hyperglycemia reduces packaging of miR-126 into endothelial microparticles^38^, while glucose starvation induces miR-126-enriches exosome formation^39^.

Consistent with the above notion, we found that exosomes derived from HUVECs cultured under glucose deprivation had increased miR-126 levels. MiR-126-enriched exosomes were shown to be transported into recipient cancer cells, where they regulated the downstream targets IRS1 and VEGF, inhibiting cell growth and transformation. Neither *EGFL7* nor *SOX2* genes were affected by miR-126. Although miR-126 is strictly expressed in ECs and excised from the *EGFL7* pre-mRNA, its expression does not affect splicing and expression of its ‘host’ gene^40^.

Recently, it has become clear that exo-miRs play a critical role in mediating cell-to-cell communication, particularly between ECs and cancer cells^39^. Upon release of their functionally active miR load inside the recipient cell, exosomal cargo can regulate gene expression via *de novo* translation and post-translational regulation of target mRNAs^8^. Several studies confirmed the presence of horizontal transfer of miRNAs via exosomes derived from tumours^41–43^. MiRs transferred by exosomes are emerging as novel regulators of cellular function, which can be used for cancer therapy. In this context, it was found that intra-tumour injection of exosomes derived from miR-146-expressing mesenchymal stem cells significantly reduced glioma xenograft growth in a rat model of primary brain tumour^44^. Another study reported that exosome-derived miR-302b significantly suppressed lung cancer cell proliferation and migration via the TGFβRII/ERK pathways, pointing to a potential target for lung cancer therapy^45^. Therefore, exosome-delivered miRs hold a substantial promise to present efficacious personalized therapeutic modalities given their use in biomarker discovery and personalized diagnostics.

## Materials and Methods

### Study population

Venous blood was collected in anti-coagulant-free tubes from NSCLC patients and healthy controls. NSCLC patients (n = 45) were recruited in the Oncology Clinic of the University Hospital of Ancona, Italy. Pathologic diagnosis was performed on pleural biopsies obtained by thoracoscopy or thoracotomy. Classification of tumour stages was based on the 7^th^ lung cancer TNM grading. The tumours were classified as squamous cell carcinomas (30%), adenocarcinomas (59%), and large-cell lung carcinoma (11%). Exclusion criteria were the presence or suspicion of an infectious disease, previous radical surgery, radiotherapy, as well as chemotherapy. The control group consisted of 31 healthy subjects recruited at the Clinic of Occupational Medicine, Polytechnic University of Marche, Ancona, Italy. Whole blood was centrifuged at 3,000 × *g* for 10 min, and aliquots of serum were collected and immediately frozen at −80 °C. All subjects filled a questionnaire including their informed consent. The study was carried out according to the Helsinki Declaration and the samples were processed under approval of the written consent statement and all experimental protocols were approved by Ethical Committee of the University Hospital of Marche, N. 51/DG 05/02/2009, Ancona, Italy.

### Preparation of exosomes and exosome-free serum fraction

HUVEC-derived exosomes enriched in miR-126 were obtained by culturing HUVEC cells in glucose-deprived media for 48 h as previously described^40^. Exosomes were isolated from serum and HUVEC in exosome-depleted serum culture medium^16^. Briefly, 10 ml serum (1:5 in PBS) or 10 ml of HUVEC exosome-depleted serum culture medium were thawed on ice and centrifuged at 10,000 × *g* for 30 min at 4 °C to remove apoptotic bodies and cellular debris. After this step, the supernatant was centrifuged at 1.6 ml of 30% sucrose/D_2_O cushion (density, 1.210 g/cm^3^) at 100,000 × *g* for 40 min. The resulting supernatant (referred to as exosomes-free serum) was collected and frozen at −80 °C until analysis. The cushion was washed in PBS and centrifuged at 100.000 × *g* for 70 min. Subsequently, the pellet (exosomal fraction) was collected, re-suspended in 200 µl of PBS and stored at −80 °C for further use. All ultracentrifugation steps were performed at 4 °C in a Beckton Dickinson ultracentrifuge with TLS-55 swinging bucket rotor. After isolation, exosomes were re-suspended in PBS, treated with 0.1 mg/ml RNase A (Qiagen) for 1 h at 37 °C to remove miR contamination, and filtered using a 0.22 μm filter before use. Protein content of purified exosomes was determined by the Bradford assay with the Bio-Rad Protein Assay Reagent (BioRad Laboratories). Exosome concentration and size distribution were determined by the NanoSight NS300 instrument. Samples were processed in duplicates and diluted with PBS to give a range of concentrations between 10^8^–10^9^ particles/ml. Samples were recorded using a camera level of 13 (camera shutter speed; 15 ms and camera gain; 350). The captured videos (60 s) were analysed using the NTA (V2.3) software (default settings) to yield particle size and concentration measurements. For *in vitro* treatments a pool (n = 4) of exosomes isolated from NSCLC patients at early (exo-NSCLC-Stage-I/II) and advanced (exo-NSCLC-Stage-III/IV) stages, and from control subjects (exo-CTRL) were used.

### Transmission electron microscopy (TEM)

Exosomes in suspension were fixed with an equal volume of 2% glutaraldehyde in 0.1 M cacodylate buffer (pH 7.4) and observed via TEM after negative staining conducted as follows: a drop (~20 µl) of the sample was left to adsorb for 1 min on a 300 mesh nickel grid coated with formvar/carbon film. The sample was then contrasted with 4 steps of drops of uranyl acetate in 3% water for 30 s. The grids were air-dried and samples observed with the CM10 electron microscope (Philips) at working voltage of 80 KV. The images of the samples were recorded by the CCD camera Veleta 130.000X, and the magnification of their diameters, expressed in nanometers, were estimated using the iTEM software (TEM Imaging Plaatform, Olympus).

### Western blotting

For immunoblotting, exosomes (10 µg protein) were resolved using 4–12% SDS-PAGE (Life Technologies), transferred to nitrocellulose membranes, and incubated overnight with anti-CD81 (generously provided by Prof. Fabio Malavasi, University of Torino, Torino, Italy). Actin (Bethyl) was used as loading control. After incubation with an HRP-conjugated secondary IgG (Sigma), the blots were developed using the ECL detection system (Pierce Biotechnology). Intensities of the individual bands were visualized by ChemiDoc using the Quantity One software (BioRad).

### Circulating miR-126 assay

MiR-126 was detected as previously described^12^. Circulating RNA was isolated by adding to 250 µL of diluted serum (1:10), exosomes-free serum (250 µl) and exosomes (20 µg protein) fractions, 750 µl of Tri-Reagent BD (Sigma); the phase lock gel (Eppendorf) was used to improve RNA recovery. To allow for normalization of sample-to-sample variation in the RNA isolation procedure, 10 μl of synthetic Caenorhabditis elegans miR (cel-miR-39) from a 6 fmol/μl stock solution was added into the denaturing solution. The miRs were further purified from total RNA using the miR isolation kit (PureLink miRNA Isolation Kit, Thermo Fisher Scientific). MiRs were eluted in the final volume of 40 μl. Reversetranscription was achieved using individual TaqMan miR Assay, and the expression quantified by qRT-PCR. To normalize the expression levels of target miR, the U6 small nuclear RNA was used as a control. Both the endogenous (U6 small nuclear RNA) and exogenous control (cel miR-39) were used for normalization and expression as 2^−∆Ct^ (endog + exog).

### Quantitative RT-PCR analysis (qRT-PCR)

Total RNA from cells was obtained using the RNeasy Mini Kit (Qiagen). The qPCR analysis of mRNA was performed using primers for IRS1, VEGF-1, SOX2 and EGFL7, and SYBR-select master mix (Life Technologies). GAPDH was used as the housekeeping gene. The qRT-PCR assays were performed using the Mastercycler EP Realplex (Eppendorf). The results were expressed as ΔCt, and fold changes in relative mRNA expression were calculated using the equation 2^−Δ(ΔCt)^.

### Cell culture

Immortalized human bronchial epithelial cells (BEAS-2B) and the NSCLC cell line (A549), obtained from the ATCC, were grown in the RPMI-1640 medium with antibiotics and 10% FBS. HUVECs were obtained from ATCC and cultured in the EGM™-Plus Growth Media following the company’s instruction (Lonza CC-2935, NJ, USA). The endothelial-like EAhy926 cells (generous gift from Jiri Neuzil, Griffith University, Southport, Queensland, Australia) were maintained in DMEM supplemented with 100 μmol/l hypoxanthine, 0.4 μmol/l aminopterin, and 16 μmol/l thymidine^46^. The cells were maintained at 37 °C in the presence of 5% CO_2_ in a humidified incubator.

### Exosome uptake evaluation

Exosomes isolated from serum of NSCLC patients or collected from HUVEC exosome-free serum culture medium were stained for RNA/DNA using acridine orange (AO, 20 µM for 1 h). AO is a membrane permeable and labels single-stranded RNAs (exo-RNAs) inside the exosomes. AO-stained exosomes were added to the culture medium of BEAS-2B and A549 cells. After 4 h incubation, the uptake was assessed by fluorescent microscopy (Axiocam MRc5 fluorescence microscope, Zeiss).

### Cell proliferation assay

Non-malignant cells (BEAS-2B) were incubated with exosomes (20 µg/ml) derived from NSCLC patients at early (NSCLC-S-I/II) and advanced (NSCLC-S-III/IV) stages and from control subjects, and cell proliferation was evaluated after 24, 48 and 72 h. In addition, an NSCLC cell line (A549) was incubated with exosomes isolated from HUVECs or glucose-deprived HUVECs, and cell proliferation was investigated. Cells were incubated with 10 µM 3-(4,5-dimethylthiazol-2-yl)-2,5-diphenyltetrazolium bromide (MTT; 5 mg/ml in PBS) at 37 °C for 3 h. After removing the media, 200 µl of isopropanol was added to dissolve the crystals. Absorbance was read at 550 nm in an ELISA plate reader (Tecan).

### Angiogenetic activity assessment

Angiogenesis was evaluated using the formation of capillary-like structures in a three-dimensional setting. Briefly, 50 μl of cold Matrigel (BD Biosciences) per well were transferred with a cold tip into a 96-well plate, 200 μl of complete medium containing EAhy926 cells (2 × 10^4^ cells per well) were re-suspended in serum-free medium (200 µl) and overlaid with Matrigel. The cells were incubated with exosomes (20 µg/ml) derived from a pool of control subjects (n = 4) and from patients NSCLC (n = 4) at early-(NSCLC-S-I/II) and advanced- (NSCLC-S-III/IV) stages. After 16 h in the incubator, the polygonal structures, made by a network of endothelial cells capillaries was established, and the images were captured at 100x magnification using the Axiocam MRc5 optical microscope (Zeiss). The tube-forming activity was estimated by counting the number of complete capillaries connecting individual points of the polygonal structures in a light microscope 24 h after transferring the cells to Matrigel. Three fields in the central area were chosen randomly in every well. The number of capillaries in control cultures was considered 100%.

### Colony-forming soft-agar assay

BEAS-2B or A549 cells were incubated with exosomes (20 µg/ml) from serum of study populations (controls and NSCLC patients at early and advanced stages), or with exosomes from HUVECs. The cells (10^4^) were then seeded in 0.7% low-melting point agar in 24-well plates, overlaid with 0.35% low-melting point agar, and cultured at 37 °C in 5% CO_2_ for 3 months. Every 7 d, 0.5 ml fresh complete medium was replaced in each well. No colony formation was found in non-exposed BEAS-2B cells used as control.

### Statistical analysis

Data are presented as means ± standard deviation (SD). Comparisons between and among groups of data were determined using Student’s *t*-test and one-way analysis of variance (ANOVA), with Tukey post-hoc analysis, respectively. A p-value ≤ 0.05 was considered significant. All statistical analyses were performed using the SPSS software.

## Electronic supplementary material

Supplementary information
