# Supplementary material for: Exosomal miR-126 as a circulating biomarker in non-small-cell lung cancer regulating cancer progression
Source: Sci Rep. 2017 Nov 10;7:15277. doi: 10.1038/s41598-017-15475-6 (PMC5681649; doi:10.1038/s41598-017-15475-6)
Supplement: Supplementary file 1 — Supplementary information [file 41598_2017_15475_MOESM1_ESM.pdf]

Exosomal miR-126 as a circulating biomarker in non-small-cell lung cancer regulating cancer progression

Franco Grimolizzi[1°], Federica Monaco[2°], Francesca Leoni[1], Massimo Bracci[2], Sara Staffolani[2], Cristiana Bersaglieri[2], Simona Gaetani[2], Matteo Valentino[2], Monica Amati[2], Corrado Rubini[3], Franca Saccucci[1], Jiri Neuzil[4,5], Marco Tomasetti [2*], Lory Santarelli[2*]

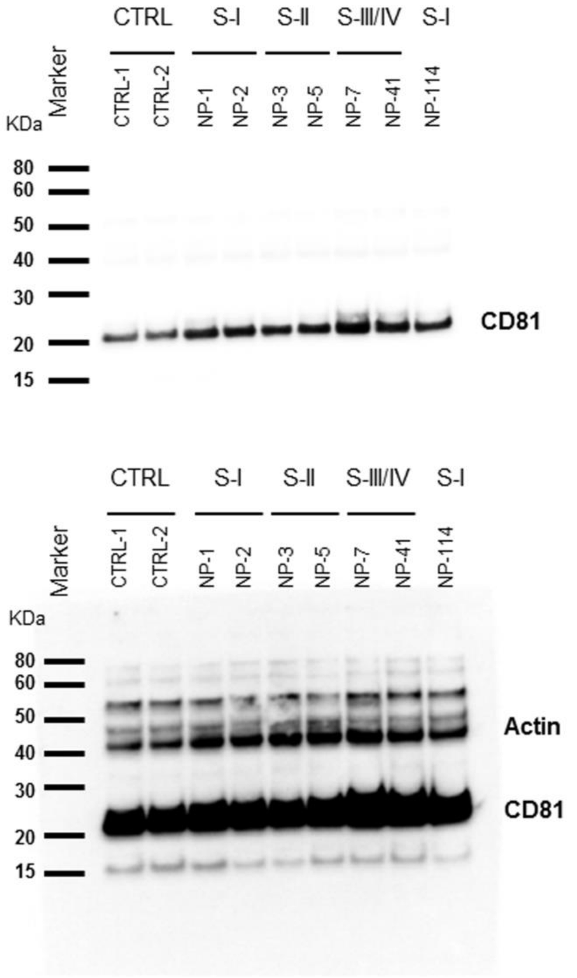

Supplementary information. Original CD81 immunoblot of isolated exosomes from controls (CTRL-1, CTRL-2) and patients affected by NSCLC at early stages, Stage-I (NP-1, NP-2, NP-114), Stage-II (NP-3, NP-5) and advanced stages, Stage III/IV (NP-7, NP-41). For immunoblotting, exosomes (10 µg protein) were resolved using 4-12% SDS-PAGE (Life Technologies), transferred to nitrocellulose membranes, and incubated overnight with anti-CD81 (generously provided by Prof. Fabio Malavasi, University of Torino, Torino, Italy). Actin (Bethyl) was used as loading control. After incubation with an HRP-conjugated secondary IgG (Sigma), the blots were developed using the ECL detection system (Pierce Biotechnology). Intensities of the individual bands were visualized by ChemiDoc using the Quantity One software (BioRad).
